# The Potato of the Future: Opportunities and Challenges in Sustainable Agri-food Systems

**DOI:** 10.1007/s11540-021-09501-4

**Published:** 2021-07-24

**Authors:** André Devaux, Jean-Pierre Goffart, Peter Kromann, Jorge Andrade-Piedra, Vivian Polar, Guy Hareau

**Affiliations:** 1 International Potato Center (CIP), Louvain-la-Neuve, Belgium; 2 Walloon Agriculture Research Center, CRA-W, Gembloux, Belgium; 3 Field Crops, Wageningen Plant Research, Wageningen University & Research, Lelystad, The Netherlands; 4 International Potato Center (CIP), CGIAR Research Program on Roots Tubers and Bananas, Lima, Peru; 5 CGIAR Research Program on Roots, Tubers and Bananas (RTB), CIP, Lima, Peru

**Keywords:** Food security, Multidisciplinary approaches, Nutrition, Sustainable intensification, Value chain, Yield gap

## Abstract

In the coming decades, feeding the expanded global population nutritiously and sustainably will require substantial improvements to the global food system worldwide. The main challenge will be how to produce more food with the same or fewer resources and waste less. Food security has four dimensions: food availability, food access, food use and quality, and food stability. Among several other food sources, the potato crop is one that can help match all these constraints worldwide due to its highly diverse distribution pattern, and its current cultivation and demand, particularly in developing countries with high levels of poverty, hunger, and malnutrition. After an overview of the current situation of global hunger, food security, and agricultural growth, followed by a review of the importance of the potato in the current global food system and its role played as a food security crop, this paper analyses and discusses how potato research and innovation can contribute to sustainable agri-food systems comparing rural and industrial agri-food systems with reference to food security indicators. It concludes with a discussion about the challenges for sustainable potato cropping enhancement considering the needs to increase productivity in rural-based potato food systems that predominate in low-income countries, while promoting better resource management and optimization in industrial-based agri-food systems considering factors such as quality, diversity of products, health impacts, and climate change effects. Research and innovation options and policies that could facilitate the requirements of both rural and industrial potato-based agri-food systems are described.

## Introduction: The Current Situation of Global Food Security and Agricultural Growth

A growing earth population and the increasing demand for food are placing unprecedented pressure on agriculture and natural resources. Today’s food systems do not provide sufficient nutritious food in an environmentally sustainable way to the world’s population (Wu et al. 2018). Around 822 million are undernourished, while 1.2 billion are overweight or obese. At the same time, food production, processing, and waste are putting unsustainable pressure on environmental resources. By 2050, a global population of 9.7 billion people will demand 70% more food than is consumed today (FAO et al. 2018). Feeding this expanded population nutritiously and sustainably will require substantial improvements to the global food system—one that provides livelihoods for farmers as well as nutritious products to consumers while minimizing today’s environmental footprint (Foley et al. 2011). A critical challenge is how to produce more food with the same or fewer resources, without increasing inequality or generating negative environmental impacts.

According to the International Food Policy Research Institute (IFPRI), the 2017 Global Hunger Index (GHI), substantial progress has been made in terms of hunger reduction for the developing world. Whereas the 2000 GHI score for the developing world was 29.9, the 2019 GHI score is 20.0, showing a reduction of 31% (Von Grebmer et al. 2019; Devaux et al. 2020). However, the current rate of progress in food supply will not be enough to eradicate hunger by 2030, and not even by 2050. Despite years of progress, food security is still a serious threat. Conflicts, migration, and climate change, and recently the COVID-19 pandemic, are hitting the poorest people the hardest and effectively maintaining parts of the world in continuous crisis. The pandemic has made the goal more elusive by creating an economic crisis, increasing food prices, and disrupting supply chains. Since the pandemic’s onset, global hunger has reached its highest level in decades and, if left unchecked, will almost certainly exacerbate the outbreak’s death toll. Recent estimates suggest that in 2020, COVID-19 will add 83 million to 132 million people, if not more, to the rolls of those without adequate food to meet their nutritional needs. In developing countries, the number of people suffering food insecurity is expected to nearly double this year, to 265 million (FAO et al. 2020).

As indicated in previous GHI reports, hunger and inequality are inextricably linked. Most closely tied to hunger, perhaps, is poverty, the clearest manifestation of societal inequality. Both are rooted in uneven power relations that often are perpetuated and exacerbated by laws, policies, attitudes, and practices. Nevertheless, the intersect of poverty with gender, age, and ethnic background among the most prominent social determinants can create critical pockets of food insecurity and extreme poverty even within moderately affected populations.

According to FAO (2002) “Food security exists when all people, at all times, have physical, social and economic access to sufficient, safe and nutritious food to meet their dietary needs and food preferences for an active and healthy life”. Food security has four key dimensions: (i) “food availability”, referring to supply; (ii) “food access”, referring to ability to produce one’s own food or buy it; (iii) “food quality and use”, referring to the level of nutrition obtained; and (iv) “food stability”, which incorporates the idea of having access at all time (FAO 2006a). This widely accepted FAO definition reinforces the multidimensional nature of food security that requires multi-sector approaches. Such approaches should combine the promotion of broad-based agricultural growth and rural development with programs that directly target the food insecure as well as social protection programs focused on nutrition, including a gender approach (Salazar et al. 2016).

Agriculture remains today the expected engine of growth for the “agriculture-based countries”, those countries with a high contribution of agriculture to GDP growth and a high share of their poor in the rural sector (World Bank 2007). Agri-food systems, defined as a socio-technical system encompassing the ensemble of interrelated social and economic actors and institutions involved in food production, processing, distribution, and consumption (Lamine et al. 2012), continue to be influenced by globalization, increased urbanization, and growing demand in urban food markets, as well as changes in consumer food preferences (FAO 2015). The role of agri-food system development has been recognized as a promising strategy for the reduction of rural poverty and transformation of the small-scale producers’ sector (de Janvry and Sadoulet 2020). Agri-food development would then translate into higher productivity among the poor, their access to markets, and to employment in the non-farm economy through industry (processing) and service provision. Therefore, enhancing food security requires policies that improve households’ ability to obtain food through production and better income. Because the potato is one of the global crops with most diverse distribution pattern (Haverkort et al. 2013) and is grown in areas with high levels of poverty, inequality, hunger, and malnutrition, it can be an effective crop for enabling smallholder families to attain food security and climb out of poverty. Hence, innovations based on potato science can be a significant vehicle for targeting the poor and hungry as part of a broader set of research and development activities.

This paper analyses first the role of the potato in global food security using the agri-food systems approach. An explicit distinction is made between two contrasting stages of food system evolution, rural and industrial food systems, and the synergies that will strengthen the potato’s contribution to global food security and income generation. It then summarizes recent data and literature describing the importance of the potato in the current global food system and the value of potato as a food security crop. The role of potato as a global food security asset is described with reference to its contribution to each of the four dimensions of food security and related to the importance and distribution of global hunger worldwide. The compiled data on global provisioning of food, feed, and other industrial use contributes to support the argument that potato-based agri-food systems present increasingly important opportunities for food security and income generation in the face of the expected trends of population growth, climate change, conflicts, migration, inequality, and the recent impact of the COVID-19 crisis. The paper analyses how potato research and innovation in rural- and industrial-based agri-food systems can enhance productivity and contribute to food security at a global scale. Focus is directed to optimizing the use of natural resources and increasing productivity while promoting better input management and optimization. The analysis leads to a list of proposed potato research and innovation options and policies to (i) enhance potato’s contribution to sustainable agri-food systems, (ii) promote better resource management and optimization with an explicit focus on more efficient production, and (iii) stress the importance of agri-food systems evolution and agriculture’s contribution to nutrition and income generation.

## Potato in a Global Food Security and Diversified Agri-food Systems Context

The potato, because of its adaptability, its yielding capacity, and its nutrition contribution, and as an important component of diversified cropping systems, has a long history of helping relieve food insecurities and contributing to improve household incomes in times of crisis and today’s population expansion. Among important issues and challenges for potato crop at global level, the European Association for Potato Research (EAPR) Conference in 2017 identified three broad concerns: (i) food security and food safety for a growing population considering consumer’s needs, (ii) sustainable and environmentally friendly production addressing the question of natural resource management taking advantage of new technologies available such as breeding techniques, biocontrol, and big data management; and (iii) innovation in practice turning scientific results into products and processes to improve the performance of agri-food systems (Andrivon 2017).

Agri-food systems development requires a comprehensive approach to address multiple issues across different levels of evolution. According to the classification proposed by the Global Nutrition Report (IFPRI 2015), there are five stages of food system evolution that reflect the stages of structural transformation that countries go through as continuums ranging from rural food systems to industrial food systems as presented below:

1. Rural food systems (low agricultural productivity, high reliance on staples (e.g. Bangladesh, Ethiopia, Bolivia)
2. Emerging food systems (more urbanized, still reliant on staples (e.g. Pakistan, Thailand, Peru)
3. Transitioning food systems (e.g. Brazil, Malaysia)
4. Mixed food systems (moderate productivity, urbanization, low dependence on staples (e.g. Germany, Italy)
5. Industrial food systems (highly urbanized, low dependence on staples (e.g. USA, NW Europe)

**Fig. 1:**
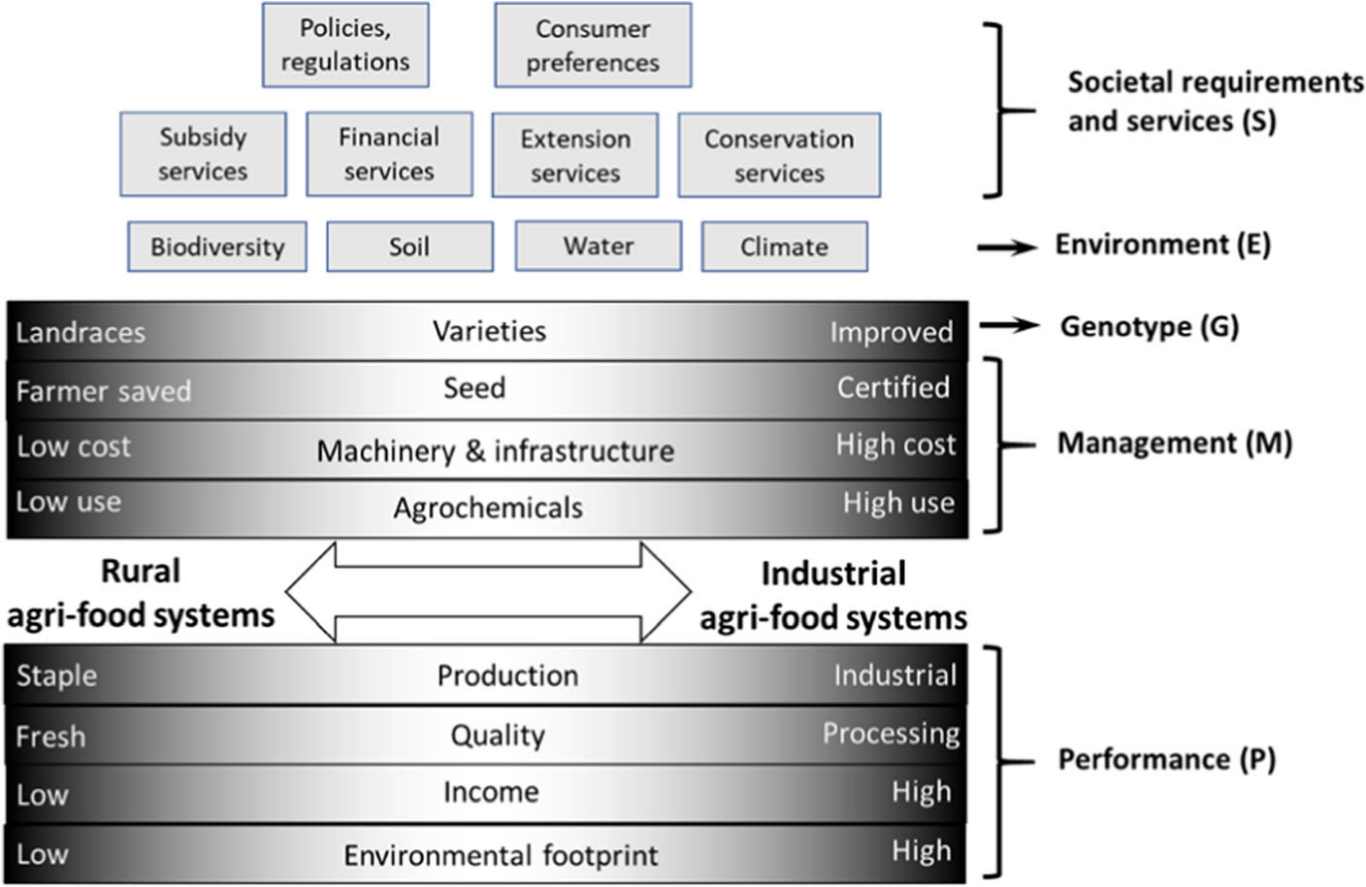
Dichotomy and performance outputs (*P*) of rural and industrial potato agri-food systems, based on genotypes (*G*), environment (*E*), management factors (*M*), and societal requirements and services (*S*) as present in Eq. (1); genotypes, management factors, and performance outputs are depicted as continuums, with extremes showing typical elements that characterize rural (left) or industrial (right) agri-food systems and combinations of both systems in-between

Firstly, we suggest using this agri-food system-based framework to analyse the potato contribution to global food security considering the dichotomy of rural- and industrial-based potato agri-food systems as presented in Fig. 1. Secondly, we propose to follow the approach suggested by Haverkort and Struik (2015) to respond to the future prospects of food security, consumers’ requirements, and food systems’ environmental footprint challenges.

This approach is expressed by the formula:

1 $$ P=G\times E\times M\times S $$

where performance (*P*) is determined by genotype or varieties (*G*), the environment or agro-ecological conditions where the crop is grown (*E*), which is evolving and will further change, the crop management and its adaptation to local socio-economic conditions (*M*), and the societal requirements and services (*S*) driven by different segments of society’s demands for food and the need to make agriculture more environmentally and consumer friendly with a focus on food safety. The actual performance of potato can be expressed as yield of fresh or dry matter per unit area or yield of the finished product per unit area recovered from the raw material after processing. These terms are also illustrated in Fig. 1.

We postulated that this performance analysis (*P* = *G* × *E* × *M* × *S*), suggested originally in the context of high-income countries, can also be used in the context of evolving agri-food systems to analyse the dichotomy between rural- and industrial-based agri-food systems. On the one hand, family farms are the backbone of agriculture in low- and middle-income countries in Africa, Asia, and Latin America. In such countries, consumers’ needs are driven by food and nutrition challenges and demand concerns, principally basic food availability in both quantity and quality from essentially local production. On the other hand, the trend in the developed world has been for many years towards intensification to achieve more outputs per unit of land, but the sustainability of this intensification is under debate especially considering agriculture’s environmental footprint (Haverkort et al. 2013). In low-income countries with mainly rural-based agri-food systems, sustainable intensification is a different challenge because it starts from a much lower level of inputs than in developed countries. This is especially the case in Africa where the potential for increasing production through area expansion is diminishing, partly due to high population growth (Headey et al. 2014). As illustration of this, Wu et al. (2018) and Jayne et al. (2014) argue that even though Africa has a high cropping intensity gap^1^, closing this gap sustainably must focus on input intensification rather than area expansion. The relevant question is how to promote technology options that allow for increased output quantity and quality (especially from the nutrition point of view), while considering agriculture’s environmental impact, preserving land and other resources in both developed and developing countries. In this context, sustainable intensification of potato cropping goes beyond production aspects and considers strong socio-economic, demographic, and environmental trade-offs to optimize performance. For example, women’s farm yields and incomes are often much lower than men’s, reflecting specific factors affecting their productivity, including limited access to land, markets, credit, inputs, technology that responds to their specific needs, and information (Polar et al. 2017; Mudege et al. 2020). In this context, it is critical to identify institutional incentives to support innovation for men and women with emphasis on research partnerships and considering multidisciplinary approaches to recognize and solve practical problems at the level of the crop, the cropping system, and the agri-food system to achieve sustainable food security in its four dimensions.

## The Potato in the Global Food System

### Potato Distribution and Consumption Worldwide

**Fig. 2:**
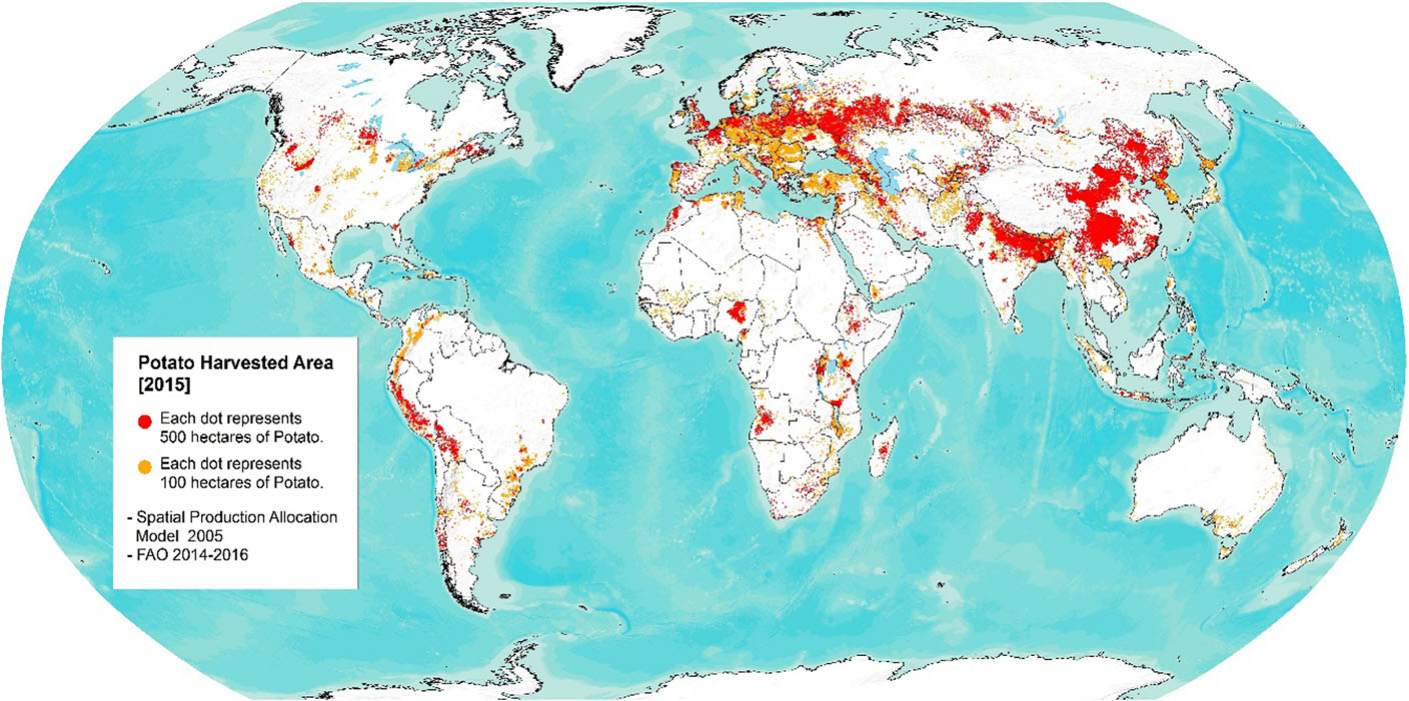
Potato distribution worldwide, harvested area (You et al. 2014; FAO 2016b)

Potato is currently grown on an estimated 20 million hectares of farmland globally, and the potato production worldwide stands for 366 million tonnes (Table 1). The highest concentrations are found in the temperate zone of the northern hemisphere where the crop is grown in summer during the frost-free period. In these regions, potato is mainly grown as a cash crop and is therefore an important source of income. In tropical regions, the crop is significant in the highlands of the Andes, the African highlands and the Rift valley, and the volcanic mountains of West Africa and Southeast Asia, where production is both for staple food and cash (Muthoni et al. 2010). In the subtropics, the crop is grown as a winter crop during the heat-free period such as in the Mediterranean region, North India, and southern China. It is only in the tropical lowlands that potato is not a main staple, largely because the temperatures in these areas are too high for tuber development and growth in traditional potato varieties (Haverkort et al. 2013). Figure 2 illustrates the current pattern of the potato distribution worldwide.

**Table 1:** Potato production indicators

Region	2016–2018			Average annual growth rate								
	Production	Area	Yield	Production (%)	Area (%)	Yield (%)	Production (%)	Area (%)	Yield (%)	Production (%)	Area (%)	Yield (%)
	(000 t)	(000 ha)	(t/ha)	1961–1963 vs 1998–1990			1988–1990 vs 2016–2018			1961–1963 vs 2016–2018		
Africa	25,026	1828	13.7	4.9	3.7	1.1	4.2	3.3	0.9	4.5	3.5	1.0
Asia*	184,937	9325	19.8	3.7	2.4	1.3	3.9	2.4	1.4	3.8	2.4	1.3
Europe	109,783	4823	22.8	−1.0	−1.8	0.8	−1.5	−2.8	1.4	−1.2	−2.3	1.1
North America	26,390	5550	47.5	1.15	−0.2	1.4	1.0	−0.5	1.5	1.1	−0.4	1.4
LAC	20,161	1051	19.2	2.2	0.0	2.2	1.7	0.2	1.5	2.0	0.1	1.9
World	366,298	17,584	20.8	0.1	−0.8	0.9	1.1	−0.1	1.2	0.6	−0.4	1.0

Source: FAOSTAT Crops, last Update June 15, 2020

*= Asia + Oceania

**Fig. 3:**
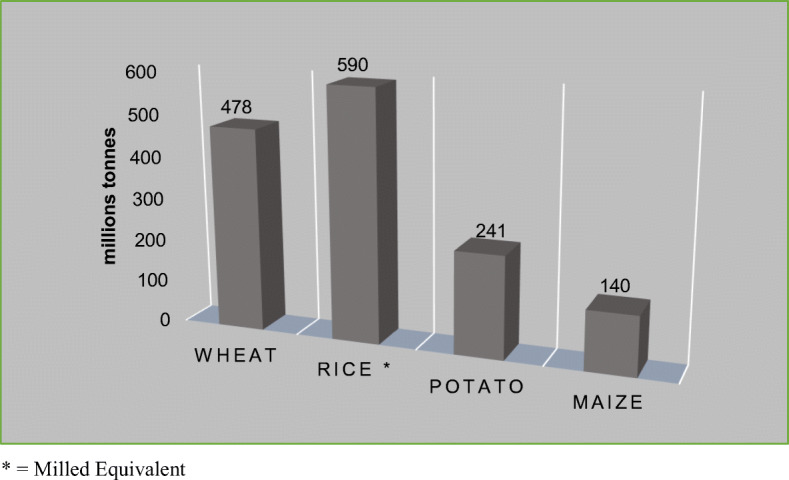
World food consumption per year for the four main nutritious crops. Asterisk indicates milled equivalent. Source: FAOSTAT, New Food Balances, last update February 19, 2020

Potato is now the world’s third most important food crop in terms of human consumption, after wheat and rice (FAOSTAT 2013) despite the large proportion of potato produce used for seed and as animal feed (Fig. 3). Consumption of fresh potatoes accounts for approximately two-thirds of the harvest and around 1.3 billion people eat potatoes as a staple food (more than 50 kg per person per year) including regions of India and China.

### Potato Production and Demand Trends by Region

**Fig. 4:**
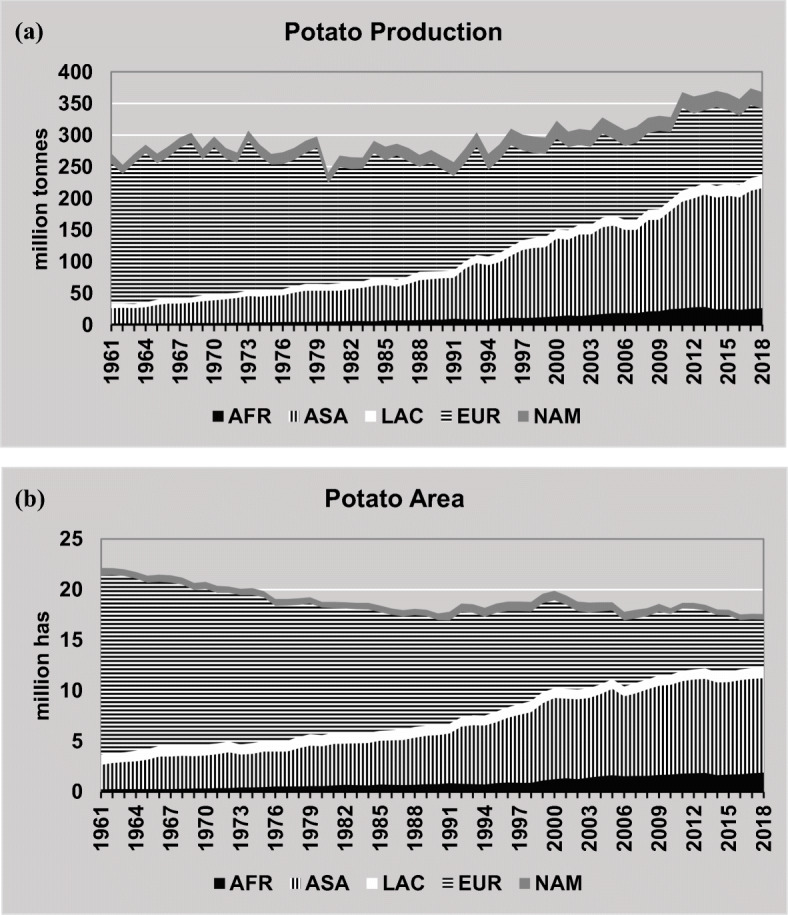
Potato supply 1961–2018. AFR, Africa; ASA, Asia; LAC, Latin America and the Caribbean; EUR, Europe; NAM, North America. Source: FAOSTAT Crops, last update June 15, 2020. (**a**) Global potato production. (**b**) Potato areas harvested

Across global landscapes, the versatility of the potato crop coupled with notable increases in production in many countries over the last two decades is unparalleled, although this increase has been mainly driven by area expansion and secondarily by yield improvements. Global statistics also indicate that potato production is shifting towards developing countries especially with strong increase in production and harvested areas mainly in Asia (ASA) and on lower scale in Africa (AFR) (especially in East Africa) and Latin America and the Caribbean (LAC) (Fig. 4 a and b). In fact, the developing world’s potato production exceeded that of the developed world for the first time in 2005 (FAOSTAT 2013). It reaffirms the increasing importance of potatoes as a source of food, employment, and income in Asia, Africa, and Latin America.

As shown in Fig. 4b, AFR zone has registered large increases of harvested area over the last 20 years, but despite the impressive growth, total production and harvested areas are still much smaller compared to Europe and Asia.

In Africa, the increase in potato production has largely been through increase of area under production, which more than doubled since 1994 and now exceeds that of the LAC region suggesting a higher contribution of the crop to local food systems. In Tanzania, for instance, potato supply has almost tripled between 2000 and 2014 (FAO 2017), while in Rwanda, potato is included in the national priority list of crops due to its role in national food security (approximately 125 kg per capita per year; FAO 2009). As world population levels are predicted to show the greatest rise in Africa in the coming decades, the increase in contribution of potato to local food systems in this region is of considerable importance (Birch et al. 2012) with high demand for potato across urban African zones and potential for production increase not only in the highlands and cool annual periods of the east, north, and south of the continent but also in the higher plateaus and mountains of Central and West Africa.

**Fig. 5:**
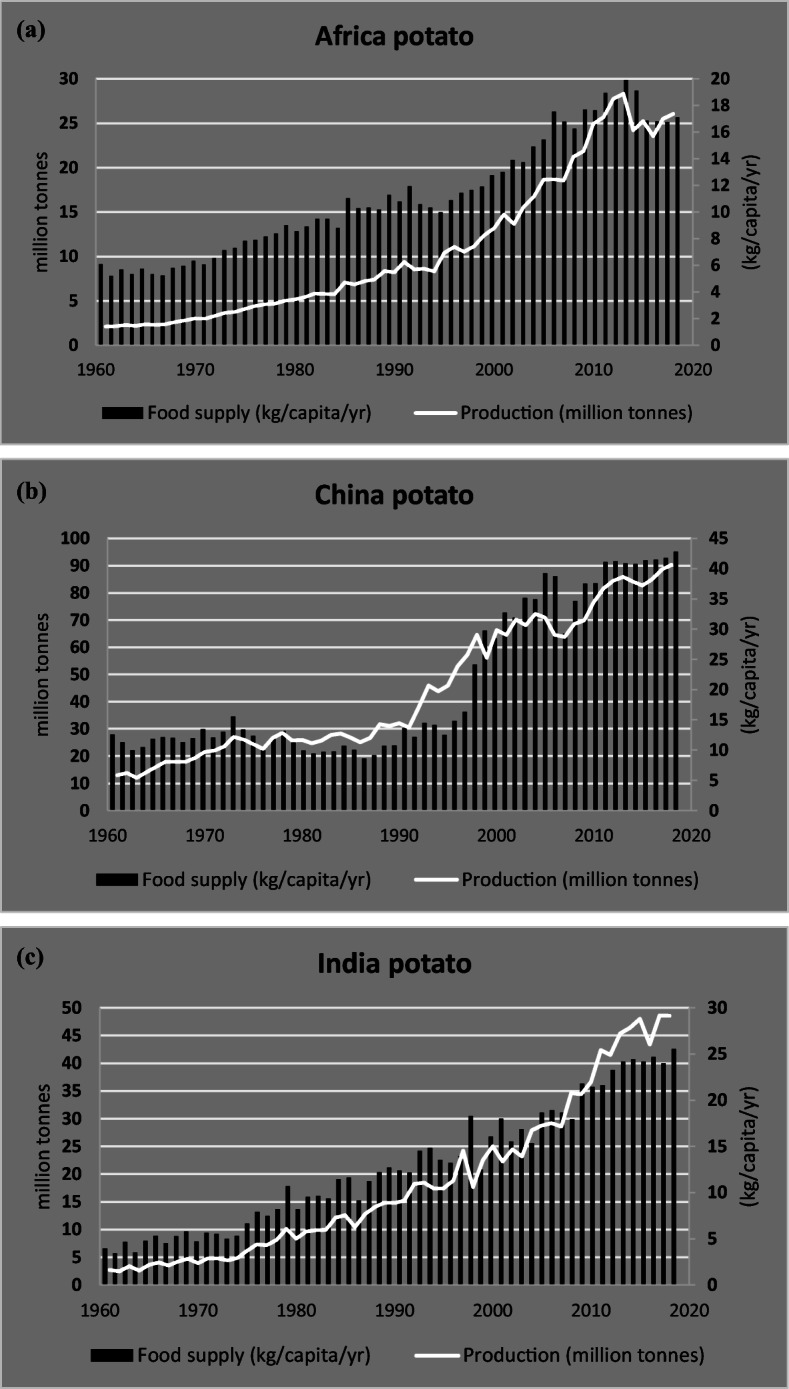
Relative development of potato production and food supply (kg/capita/year) in Africa (**a**) China (**b**), and India (**c**). Period 1961–2018. Source: FAOSTAT, Crops last update June 15, 2020, and Food Balances last update February 19, 2020

Asia, China, and India have experienced nearly a half century of steady growth in potato production (Fig. 5 b and c). Both countries also have ambitious growth targets for future years. For some decades now, the Chinese state has been working to increase national potato consumption, also launching a campaign since 2014 to promote both the cultivation and the consumption of this tuber (The Wall Street Journal 2015). China became the world’s largest potato producer in 1993 and currently accounts for almost one quarter of global potato production and about 28% of total cultivated areas (FAO 2017). Potato in China is mainly used for food, both as a vegetable or in processed forms, while a smaller part is also consumed as animal feed (Scott and Suarez 2012a). Potato in India is mainly grown in the Indo-Gangetic plain, either as monoculture or in rotation with maize, wheat, and/or rice, and it is regarded as both an important staple and a cash crop. Following the growth in production volumes, potato yields in India have also increased significantly, at an average of 2% per year, because of successful breeding programs, quality seed systems, and storage infrastructure that have reduced post-harvest losses (Scott and Suarez 2011). Looking at other Asian countries, such as Bangladesh and Pakistan, the potato production has also greatly expanded during the last decades (Scott and Suarez 2012b).

Potato production in LAC has increased over the past 60 years (Fig. 4a); the annual average potato domestic supply has increased from 7.2 million tonnes in the 1961–1963 period to 19.6 million tonnes in 2011–2013, which represents an average annual growth rate of 2%. By way of comparison, growth rates for potato production in ASA and AFR averaged over 4% for a similar period, i.e. more than double those of LAC (Scott 2011). Most of the production is oriented towards human consumption (74%, maintaining this trend throughout the period), and it highlights a relatively low processing level of 1% (FAO 2017).

The role that potato plays in the diets in LAC is variable including (a) basic staple, producer/consumers in the Andean highlands, (b) complementary vegetable for urban households in most of South America, (c) relatively expensive complementary vegetable in much of Central America and the Caribbean, and (d) a popular fast food in the form of French fries in urban markets throughout the region (Scott 2011). Per capita consumption of potatoes in LAC increased slightly from 22 on average between 1961 and 1963 to 25 kg/person between 2011 and 2013. But these regional trends do not reflect the important differences in trends at the sub-regional and country levels. Peru, for instance, is one of the countries where potato consumption has grown significantly, reaching in 2015 a figure of 85 kg/person. This is due to various public-private policies, rural infrastructure development, expansion of supermarket trade focused on potatoes, and a strong relationship with the gastronomy sector promoting Andean food including the native potato and its products. Brazil and Mexico have increased their consumption, although their absolute values, 18.5 and 14.8 kg/person, respectively, remain low compared to other countries in LAC.

The USA is the fifth largest potato producer in the world with more than 422,000 ha harvested in 2017 and a total output of almost 20 million tonnes (Fig. 4a). Although in the USA potato is no longer the traditional staple of the past, it is nevertheless gaining increased appreciation by nutritionists because of its nutrient density, which means that for each calorie of potato eaten there is an ample return of essential nutrients and its contribution to a more balanced diet. The per capita potato consumption in the USA averages only a little more than 100 cal a day but provides a significant amount of the needed vitamin C and other essential vitamins and minerals (Bohl and Johnson 2010). There is also a large demand by the processing industry for producing commodities like frozen French fries and chips for both the local and foreign markets. Potato yields in the USA have more than doubled over the last 50 years, rising from 22 in 1961 to 49 t ha^−1^ in 2016. This increase in yields has been suggested to be primarily driven by improvements in management rather than genetic improvements, since most breeding programs have traditionally focused on quality traits such as dry matter content and storage longevity to meet the demands of the processing industry and the consumer (Douches et al. 1996).

**Fig. 6:**
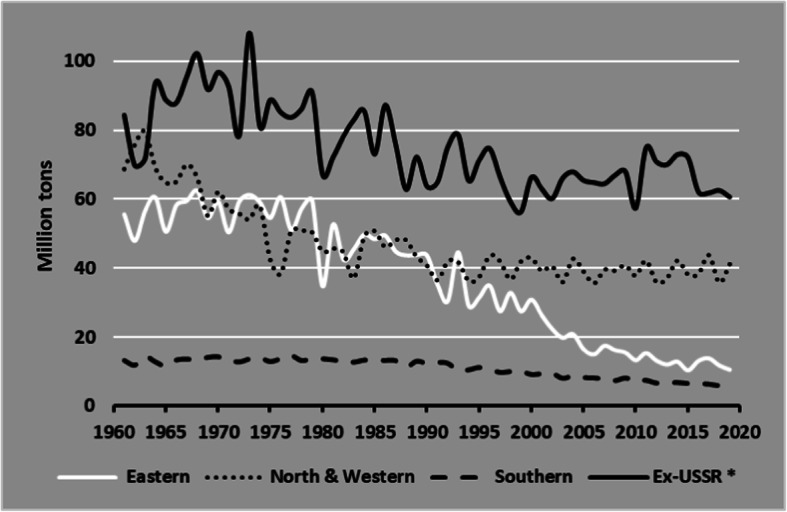
Potato production in Europe and Ex-USSR* 1961–2019. Source: FAOSTAT Crops, last Update December 22, 2020. *Ex-USSR (include European and Asia countries)

Five Northwestern countries (Germany, France, The Netherlands, the UK, and Belgium) constitute together the strongest potato producers in Europe (Fig. 4 a and b), due to potato yields higher than 40 t ha^−1^ in this area and to the strong links of production with the dynamic European potato processing industry (Fig. 6). Potato is also prevalent in Eastern European countries, particularly in Russia, Ukraine, and Poland where per capita consumption has traditionally exceeded 100 kg annually. Although Eastern Europe constitutes the region with the highest use of potato as animal feed globally, feed use of potato has been steadily declining over the last 20 years and being replaced by cereals, most notably in Poland. This decline in feed use, together with the shift of diets towards low-calorie food and a trend to spend less time on cooking, as observed in Western European countries, has led to a significant decrease in demand for fresh potatoes, and therefore potato production in the continent has been falling. Figure 6 illustrates the decline in potato production in Eastern Europe and the former USSR countries, while in North West Europe, production and harvested areas were stabilized from 1990 up to now mainly under the driving force of the potato processing industry.

**Fig. 7:**
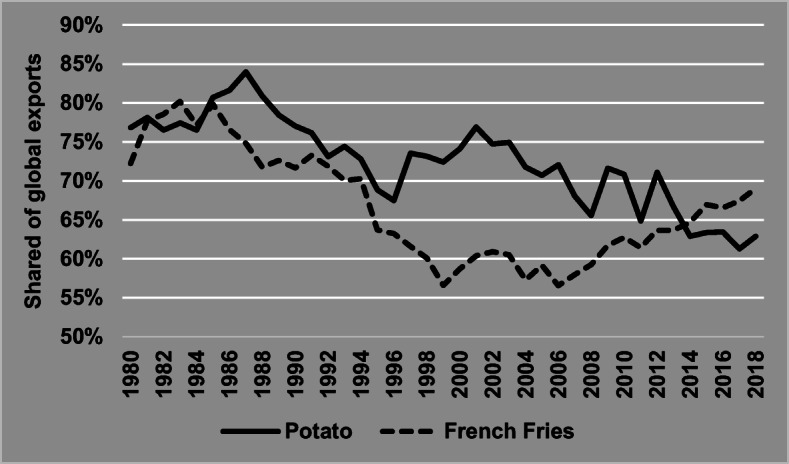
European share of global exports for potato (fresh and seed) and French fries 1980–2018. Source: FAOSTAT, TRADE, last Update August 26, 2020

Countries like France, Denmark, and Belgium have increased production over the last decade, due to growth of the processing industry (French fries, crisps) and starch production (Eurostat 2017). Moreover, the competitiveness of the potato industry has established Europe as the world’s biggest net potato exporter, accounting for more than 60% of all exports of fresh potato and a similar percentage of global exports of French fries (Fig. 7). These statistics concern mainly intra EU trade, but also export of seed potato to non-EU countries, primarily to Mediterranean African countries like Egypt and Algeria (FAO 2017).

Tables 1 and 2 give a synthesis of the potato indicators by region confirming the expansion of potato in ASA zone that is now the major potato producer continent. In AFR zone, the potato growth rate has also been strong with Egypt, Malawi, South Africa, Algeria, and Morocco producing more than two-thirds of all the potatoes in the region. In many countries of LAC zone, potato areas have declined although output has risen due to improvements in productivity. Potato production growth rate in EUR and NAM zones has declined due to a significant decrease in demand for fresh potatoes that was partially compensated by a large demand from the processing industry as described before. In NAM, yields increased rapidly between 1961 and 1990 and then somehow stagnated, suggesting that yields are near their potential in the region and there may exist genetics limits. In EUR zone, on the contrary, the relative larger yield growth rates occurred after 1990.

**Table 2:** Potato utilization, consumption, and trade indicators

	Utilization					Consumption	Trade	
Region	Domestic supply (%)	Food (%)	Seed (%)	Feed (%)	Other uses (%)	Food supply quantity (kg/capita/yr)	Export Quantity (000 t)	Import Quantity (000 t)
Africa	100	74.8	6.7	4.0	14.5	16.9	1043	1007
Asia*	100	68.2	4.5	11.9	15.3	29.2	2795	5188
Europe	100	51.3	14.7	18.6	15.4	78.3	21,031	15,662
Northern America	100	85.0	5.6	0.5	8.9	53.2	5491	3221
LAC	100	72.0	5.5	7.7	14.8	23.0	499	1704
World	100	64.7	7.9	12.5	14.9	33.1	30,859	26,782

Source: FAOSTAT, New Food Balances, last Update February 19, 2020

*= Asia + Oceania

**Fig. 8:**
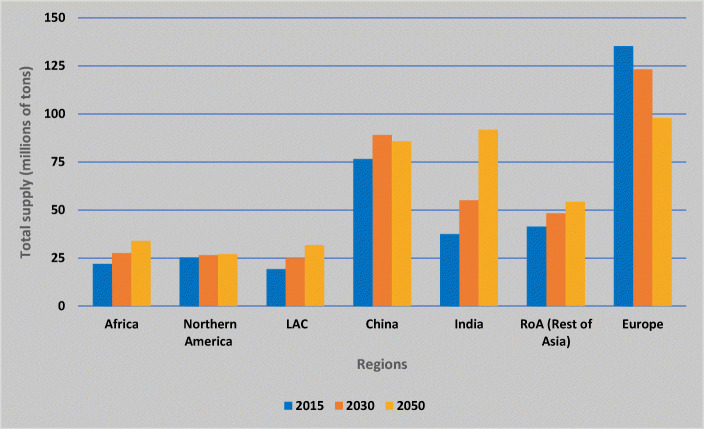
The future of potato production adapted from Rosegrant et al. 2017

Future trends by region indicate a major increase in ASA and AFR zones as compared to other regions (Fig. 8). Considering some underlying assumptions such as population growth, climate change, and economic growth pathways, the UN projects a population decline in China, not to mention growth per capita GDP which will affect diet composition. Therefore, the future supply of potato in China will not continue to grow as fast as in the past. According to Rosegrant et al. (2017), it is in India where potato supply will almost triple because of the very high population growth, especially under certain socio-economic scenarios.

## The Potato in the World’s “Nutrition Transition”

### The Dual Role of Potato in the Economy of Family Farming

The potato continues to play a dual role in the economy of family farming: firstly, by providing food for home consumption for low-income men and women who rely on agriculture for subsistence as well as income. It therefore plays a role in diet diversification in many developing countries where family agriculture and smallholders continue to supply local markets and access fresh and affordable agricultural produce.

Secondly, as food systems are changing rapidly, the potato also plays an important role in a “nutrition transition” global context. The “nutrition transition”, defined as the shifts from traditional towards western type diets, is driven by globalization, the emergence of fast-food outlets and supermarkets, and the rising levels of income and is led by major shifts in the availability, affordability, and acceptability of different types of food including processed and prepared potatoes (Gillespie et al. 2017). Higher income and increased urbanization have led to increased demand for processed potatoes; the urban poor and emerging middle-class households tend to reduce their consumption of cereals, roots, and tubers while increasing for processed, convenience/fast foods at supermarkets, restaurants, and informal street foods. This trend presents opportunities to increase the income of the numerous small family farms in rural areas where potato can present additional economic benefits compared to other crops of the food system. For example, in South Africa, potato consumption has been growing in urban areas as part of the staple food consumption of the middle class, although maize remains the primary staple in rural areas. In parallel, the emergence of this new trend increases the need for labour and thus generates income opportunities for women and young people who have limited or no access to land and other productive resources.

Nevertheless, potato is still an important staple in rural food systems and in emerging food systems which are urbanized but where consumers still rely on staples such as potatoes. On the contrary, in industrial food systems, highly urbanized as in Northwestern countries in Europe and North America with the development of the processing industry, there is a very low dependence on traditional staples (Gillespie and Van den Bold 2017). These different stages of food system evolution highlight the need to articulate different strategies to enhance agriculture’s contribution to diet quality and nutrition for each typology. They also imply the existence of a dichotomy between the strategies required to develop technologies and production methods to respond to the requirement of the rural and industrial food systems. As highlighted by Andrivon (2017), the broad challenges remain similar such as ensuring food security and food safety and sustainable and environmentally friendly production, but there is a need for more exchange, knowledge sharing, and collaboration between researchers in the North and the South for enhancing the research capacity of the South. On the other hand, knowledge acquired in some rural areas regarding potato production (e.g. usefulness of biodiversity of local wild potato species to better control abiotic and biotic stresses) can also be useful to enhance potato production in industrialized countries. It is also important to stress the necessity to make research results available and adapted for turning scientific results into products responding to the needs of both rural and industrial agri-food systems.

Potato also remains a food security crop because, as mentioned by Haverkort and Struik (2015), potato, mainly fresh potato, used to be a “local for local” crop, and it still is in many developing countries because of the bulkiness and the limited storability of the seed and ware tubers. The comparatively short maturity period, nutritious characteristics, adaptability to various climate conditions, and employment and income opportunity that characterize potato make it a resilient crop that can secure vulnerable livelihoods under the effects of climate change and changing market environments. Compared to other staples, and except for potato dedicated to be processed as it is mainly in industrial countries, fresh potato is a thinly traded commodity in global markets and is absent in major international commodity exchanges. It is therefore subject to less price volatility at global scale. Thus, fresh potato can be relied upon to smooth the disruptions in global food supply and demand that have an impact on other commodity prices, such as witnessed during the 2007–2008 and subsequent food price spikes (FAO 2009).

### Potato, Nutrition, and Health

Today, potato is consumed globally; it plays an important food and nutrition security role in many countries of the South, while in the North, consumer demand is for better tasting and healthier convenience food. Potato can be promoted as a healthy and versatile component of a nutritious and balanced diet including other vegetables and whole grain foods. From a human nutrition perspective, potatoes are an essential source of energy, protein, and micronutrients like iron and zinc. They also provide key nutrients to the diet including vitamin C, potassium, and dietary fibre. Likewise, in developing countries, it contributes to combat micronutrient deficiency, also referred to as hidden hunger, that is a major global public health problem affecting an estimated 2 billion people globally (Bailey et al. 2015). Improving the phyto-availability of iron and zinc from potato varieties in order to fortify the tubers for human consumption to reduce global micronutrient malnutrition can be achieved by breeding and agronomic fortification (Kromann et al. 2016; Amoros et al. 2020; Jongstra et al. 2020). When eaten with its skin, a single medium-sized potato of 150 g provides nearly half the daily adult requirement (100 mg) of vitamin C. It is also a good source of vitamins B1, B3, and B6 and minerals such as iron, potassium, phosphorus, and magnesium and contains folate, pantothenic acid, and riboflavin. As an example, potato plays an important role in the food basket in Andean countries such as Bolivia, Ecuador, and Peru where it continues to make up a relatively high proportion of daily calorie availability, reflecting its importance as traditional source of energy. Native potato varieties are commonly grown in the Andes and are a significant part of the local diets. Some of these native varieties have higher contents of micronutrients (Zn and Fe) and are rich in antioxidants.

It should be mentioned that potatoes have been related to increased risks of obesity mainly because of their high glycemic index. Recent reviews of clinical intervention and observational studies centred on the potato concluded that these studies did not provide convincing evidence to suggest an association between intake of potato and risks of obesity, type II diabetes (T2D), or cardiovascular disease (CVD) (Borch et al. 2016). However, as part of the trend towards urbanization and associated lifestyles, raising incomes, and greater consumption of “convenience foods”, demand for fried potatoes is increasing. Overconsumption of these high-energy products with reduced physical activity can lead to overweight and obesity. Therefore, the role of fried potato products in the diet must be taken into consideration in efforts to prevent overweight and diet-related non-communicable diseases, including heart disease and diabetes. For these reasons, even though the potato is a nutritious staple crop, it has often been associated to a meal component with no specific attributes in many societies, even in developing countries.

Another aspect to be considered in the case of fried potato (mainly processed French fries and potato crisps under temperatures higher than 100 °C) is the presence of acrylamide that has been a matter of debate since its finding in 2002 (Pedreschi 2007). This component has been classified as probably carcinogenic to humans, with significant neurotoxicological effects. Acrylamide formation results from the Maillard reaction in the presence of reducing sugars (glucose and fructose) and the amino acid asparagine as precursors. There are two ways recognized to mitigate acrylamide formation: either by removing its precursors from raw potatoes through selecting relevant potato varieties and mainly management of fertilizer use, harvest time, and storage conditions or by applying processing methods to inhibit or reduce the intensity of the Maillard reaction (Zhang and Zhang 2007). Research efforts still need to be developed to reduce acrylamide formation in fried potatoes (Israilides and Varzakas 2015). However, it remains a huge challenge for the processing industry as reducing sugars are also important components to reach desirable sensorial properties in the final products such as flavour, smell, colour, texture, and taste of French fries or crisps.

## Policies and Strategies for the Development of the Potato as a Food Security Crop in Developing Countries

The role potato can play as a food security crop at national scale has been addressed in some developing countries with different policies, either sectoral and crop-specific or at the macro level. In its quest to improve food security for a rising population, the Government of China is developing a national plan to increase production and consumption of potato and promoting the crop as a staple instead of a vegetable. In 2016, the Ministry of Agriculture released a guideline for potato as a staple, targeting around 6.7 million ha of planting area and 120 million tonnes of production in 2020, of which 30% was for processing. Many provinces, thus, began encouraging potato processing for staple food and subsidizing processing as bread and noodles, investing around 13–26 million euro within a 3-year period (Xiaoping Lu pers. comm. 2020). This status can give access to important complementary policies and resources at national and regional level and to subsidies from the central government. It also recognizes the double role of potato in current China where potato is still a major staple for poor rural areas where local governments continue to provide subsidized inputs (e.g. clean seeds of selected varieties), while at the same time being at the forefront of an increasingly private sector-led processing industry, accompanied by rising incomes in urban populations and diversification of diets (Scott and Suarez 2012a, 2012c). Potatoes can create opportunities for value addition and employment for women especially in post-harvest and processing in rural areas, because of their perishable and bulky nature. Extensive transformation of current technology will be required to capture benefits from transformation and processing of potatoes for growing urban populations. However, unless gender roles and needs are considered, innovation can worsen gender inequality (Sarapura 2012). Increasing equitable access and benefits from technology for women can increase productivity and reduce gender disparities (FAO 2014).

In Peru, the major centre of origin of the potato, a large effort began in the early 2000s to develop a competitive and inclusive native potato value chain for domestic markets. Initially led by the International Potato Center (CIP), the initiative gathered several private and not-for-profit actors to add value to the native potato grown by small farmers while developing a niche market. Several new products were developed in the process, for example, selected native potato varieties for fresh consumption sold as gourmet potatoes in innovative packages in large supermarket chains, snacks such as coloured native potato chips, and culinary innovations in the gastronomy sector featuring native potato as a central component of sophisticated dishes. The innovations in the value chain continued, and rounds of new products emerged, including frozen native potato fries, native potato-based liquors, and even cosmetics made from potato. Although no specific sectoral policies were behind this initiative, the development of the native potato value chain took advantage of government policies at the macro level promoting private sector and market-led developments, the fast growth of Peru’s economy, and the increased purchasing power of the population since the beginning of the twenty-first century. While the Government of Peru focused on public investments to promote export-oriented agricultural growth, the experience with the native potato value chain has proven successful to link small potato farmers to domestic markets and to develop a more inclusive growth strategy of the highly diverse agricultural sector of the country (World Bank 2017).

A final example of policies that have been adopted to promote the potato sector in developing countries is through seed laws and regulations. Seed degeneration due to viruses is one of the most common constraints affecting potato productivity. In most developing countries, however, seed systems were first established following developed country standards for cereals and grains, promoting the use of certified seed under a formal seed system. This has led to very low use (less than 10%) of certified seed potato in most developing countries. To increase access to quality seed by smallholder farmers, the Food and Agriculture Organization (FAO) of the United Nations promoted the definition of a new seed category, the Quality Declared Seed (QDS), that relaxes some of the standards required for certified seeds and recognizes the importance of seed producers in providing seed of enough quality through the informal seed system (FAO 2006b). This is particularly important for indigenous populations of the high Andes that produce native potato varieties that are not even registered in the formal seed system and are not produced by commercial suppliers. Similarly, women who traditionally play an important role in seed management and storage often operate in the informal sector maintaining their own seed and promoting access through local trade schemes (Mudege et al. 2020). Ethiopia adopted the QDS definition in a new seed law passed in 2016 without distinction of crops. Peru (in 2018) and Ecuador (in 2013) have modified the seed regulation for potato to accept the use of QDS. However, differences still exist on how countries are beginning to adopt this category. While in Peru, the new regulation defines the category as QDS similar to the FAO definition, in Ecuador the regulation defines the new category as “common seed” and introduces aspects of the FAO definition for QDS. Other countries are updating the regulations regarding seed quality assurance systems for potato to increase availability and access of quality seed by farmers. A broad range of changes are proposed, from relaxing some of the standards required for certified seed to allowing the use of private inspection services to increase the number of seed producers that can be inspected each season (e.g. Kenya). One of the motivations of these changes is the increasing recognition of the role of the potato crop for national food security and the need to legalize processes that can bridge the gap between informal and formal seed production with the aim to foster support for economically sustainable quality insurance in seed potato production. Some concerns have been raised, however, on the potential consequences of relaxing seed quality standards on the incidence of seed borne pests (e.g. *Ralstonia solanacearum*, *Globodera pallida*), pointing out the need of rigorous evaluation and adaptation of QDS and similar approaches to local conditions (Forbes et al. 2020).

## Opportunities and Challenges in Potato Research and Innovation

### Potato Production in Rural- and Industrial-Based Agri-food Systems

**Fig. 9:**
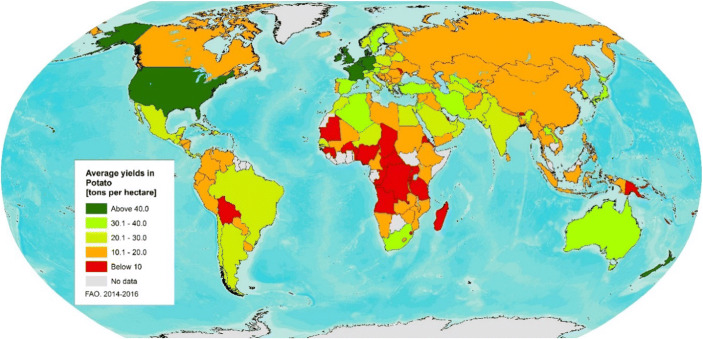
Global distribution of potato yields (tonnes ha^−1^), FAOSTAT, 2014–2016

Potato’s support to food availability can be achieved through improved productivity, either by increasing yields or expanding production areas, combined with technologies that reduce post-harvest losses. Figure 9 shows the global distribution of yields and the low yield levels in most of the developing world, where actual yield observed is usually much lower than the attainable yield. Actual yields range from below 5 to 10 t of fresh tubers ha^−1^ in rural agri-food system areas (median yield in Uganda: Gildemacher et al. 2009) to well above 40 up to 100 t fresh tubers ha^−1^ in industrial agri-food system areas (in Columbian Basin, USA: Kunkel and Campbell 1987).

The yield gap, expressed as the difference between actual yield in farmers’ fields and the attainable yield—using best agricultural practices—leaves a great potential for improvement considering that, in developing countries, the full expression of the crop’s yielding capacity has not yet been achieved. Much improvement is needed in agronomic practices, quality seed production, and varieties tolerant/resistant to abiotic and biotic threats (Birch et al. 2012). The high nutritional value and the actual performance of potato mentioned before in industrial-based agri-food systems reinforces the potential of potato to respond to food security challenges, also, in developing countries. On the other hand, lowering the environmental footprint mainly in intensive potato production systems will require actions on the use of agro-chemical inputs (fertilizers and pesticides) in production as well as in storing facilities and on optimized management of natural resources. Finally, as described in the “Potato in a global food security and diversified agri-food systems context” section, the societal or consumer requirements will vary according to the economical context. In high-income countries, consumers are looking principally for healthy and easy to prepare foods at an affordable price while in the developing world where rural-based agri-food systems are common, consumer needs are driven by the food and nutrition challenges related to basic security of food availability, access, and stability.

### Research and Innovation in Sustainable Potato-Based Agri-food Systems

Considering the large differences between rural- and industrial-based agri-food systems (Fig. 1), locally adapted research and innovation pathways seem mandatory. Research and innovation options need to respond to a variety of food systems and stages of food system evolution to strengthen potato’s contribution to global food security and income generation and to reduce potato’s environmental footprint mainly in industrial agri-food systems. Identification of the most important constraints and proper selection of target areas for potato research and innovation, together with identification of potential synergies among potato-based agri-food systems and common development objectives, is expected to speed up the innovation process, as will optimized scientific and technical knowledge transfer among potato-based agri-food systems worldwide. For instance, considering the climate change effects, many simulation exercises based on IPCC (Inter-governmental Panel on Climate Change) scenarios and biology models are underway and suggest that future potato cropping systems could differ from those we know today with the implication that new varieties will be required to respond to these new conditions (Andrivon 2017; Quiroz et al. 2018).

Sustainable potato production and efficient use of resources will require adjustments and redesigns of the current cropping and processing systems. In such a context, two main options can be considered to increase food security and income generation and to reduce the environmental footprint: (i) produce more with less through better input management and optimization and (ii) produce just as much but waste less, both before and after harvest through better value chain management, better storage, processing, and marketing operations and responding to increased involvement and awareness of consumers (Andrivon 2017).

#### Identification of Potato Research and Innovation Options

As an attempt to analyse how to combine and score different research and innovation options according to their effect on sustainable agri-food system indicators and their relation to the four dimensions of food security, Table 3 suggests a list of key priority research and innovative technology options in the spheres of the terms G, M, and S of the performance equation (see “Potato in a global food security and diversified agri-food systems context” section). The authors scored key options using some critical indicators of sustainable agri-food system intensification related to productivity, agricultural income, human wellbeing, and environmental sustainability according to Smith et al. (2017). We scored key research and development options using a simple scale of high, medium, and low effects according to their expected relation (according to literature) to sustainable intensification indicators (efficiency in use of water, land, nutrient, and pesticide; farmer incomes; efficiency in nutrient supply; reduction in environmental footprint) and their main contribution to one of the four dimensions of food security. After comparing the individual scores and discussing the reasons for differences in scores, the final scoring was adjusted.

**Table 3:** Qualitative evaluation of key research options to enhance productivity and food security dimensions through the performance of potato production systems (expected trends based on the authors appreciations and the literature related to indicators of contribution to sustainable agri-food systems intensification)

Key research options to enhance the performance of potato production systems	Food security dimensions						
	Availability				Access	Use/quality	Stability
	Indicators of contribution to sustainable agri-food systems intensification						
	Water use efficiency	Land use efficiency	Nitrogen and phosphorus use efficiency	Pesticide use efficiency	Farmer’s income	Increase in calorie and nutrient production efficiency	Reduction of environmental footprint (soil, water, air)
Breeding and variety development (G - Genotype)							
*High yield potential (considering low input of water, nitrogen, and phosphorous)*	**	***	***	Neutral/negative. High-productive crops might need additional pesticides	***	**	
*Pest resistance (*e.g. *late blight, viruses)*		***	*	***	**	**	***
*Tolerance to drought/heat/salinity*	***	***	*		**	**	*
*Earliness*	***	***	***	**	***	***	***
Biofortification (e.g. Fe and Zn)					*	**	
*True hybrid varieties (diploid F1 hybrids for TPS) and gene editing*	***	***	***	***	**	***	***
Potato seed production (M - Management)							
*High quality seed production and distribution*		**	*	*	***	*	Neutral/negative. High-quality seed use might motivate increased chemical inputs
*Farmer-based seed production*		*	*	*	**	*	**
Decision support tools (M - Management)							
*Decision support and diagnostic tools for pest control*		*		***	**		***
*SMART agriculture approaches (sensor use for precision in N,P,K, pest control and water use)*	***	**	***	**	**	*	***
Efficient use of sustainable resource (M - Management)							
Ecosystem management and biodiversity use	*		*	**	* Policy dependent	*** Use of potato biodiversity can increase nutrient production efficiency	***
*Soil and water management*	***	**	**	*	**	***	**
Efficient and inclusive value chains (M - Management and S - Societal requirements and Services)							
Value chain innovation	*	*	*	*	***	*	**
Post-harvest management (M - Management and S - Societal requirements and Services)							
*Post-harvest losses assessment and reduction*	*	*	*	*	***	**	***
*Standards of quality tubers for fresh markets and processing*	*	*	*	**	**	***	**

Suggested key research options that can enhance the performance of a wide range of potato agri-food systems are in italic

Expected trends: ***, high effect; **, medium effect; *, low effect; when the cell is blank, it means “no expected effect”

Considering breeding and variety development, improvement in nutrient efficiency, pest resistance, tolerance to drought and heat, as well as earliness and true hybrid varieties are expected to contribute to enhanced sustainable productivity and food security in rural as well as industrial potato-based systems. Tolerance to salinity and biofortification could be mainly dedicated to rural systems in developing countries.

Regarding seed potato production, high-quality seed production and distribution is expected to enhance productivity in rural-based system but also in industrial-based systems with more local production where local agro-ecological conditions permit quality production. That is mainly to limit the economic and environmental costs of long-distance transport and to reduce the risks associated with the spread of pests through tuber seed (e.g. quarantine bacteria or nematodes) even at very low level in certified seeds. Also, quality farmer-based seed production needs to be improved in rural systems in low-income areas.

Development of decision support tools and precision farming practices are expected to serve both rural- and industrial-based production systems through improvement of control of pests on the one hand and improvement of water and fertilizer efficiency on the other. These are typical research options for which technology adaptation from the North to the South can contribute to global food security. For example, the introduction of selected early potato varieties has contributed to intensification of cereal-based systems in rural agri-food systems in India (Arya et al. 2015).

The efficient use of natural resources, efficient and inclusive value chains, and post-harvest management are topics that still need more consideration and improvement in both rural- and industrial-based agri-food contexts.

A conducive enabling environment is crucial to harness the potential of new technological innovations and foster a thriving local potato sector. The potential contribution to productivity enhancement and food security of any of the G and M technologies or development approaches, as expressed by the performance Eq. (1) (*P* = *G* × *E* × *M* × *S*), is linked not only to the external factor (*E*) agro-ecological environment but also to a strong (*S*) factor, the societal requirements, and services (Fig. 1). Positive effects will be strongly influenced by the local context where they are implemented, e.g. policies. Financial and non-financial services are key components for achieving efficient food systems, such as roads, and access to technical and market information and other productive infrastructure (de Janvry and Sadoulet 2020).

The next section “Producing more with less through better inputs management and optimization” attempts to respond to the above-mentioned G and M factors, linking potential research and innovation options to the current local and global situation of potato agri-food systems considering the option suggested previously “Produce more with less through better inputs management and optimization”. The “Produce just as much but waste less through value chain development and better post-harvest management” section reviews the importance of value chain development and post-harvest management to improve the efficiency and profitability of potato-based agri-food systems responding to the evolving food systems context and the demand from consumers, considering the second option “Produce just as much but waste less”.

#### Producing More with Less Through Better Inputs Management and Optimization

##### Potato Breeding, a Driving Force Towards More Efficient Potato Production

With the recent discoveries on the potato genome sequence (PGSC 2011) and the possibilities occurring with new breeding technologies (NBTs), potato breeding appears as the number one opportunity to improve potato production for global food security (Birch et al. 2012). For genotype development (G), priority should be given to achieve a combination of traits to enhance stress tolerance and nutritional aspects to better respond to contextual evolving changes, especially climate and local needs. The current development in participatory breeding and demand-led and gender-responsive product profile development is helping to better define the crucial trait combinations required, which will facilitate acceptance of new genotypes by growers and other stakeholders (Schulte-Geldermann et al. 2012; Ashby and Polar 2019).

The development of early and high yielding varieties with resistance to *P. infestans* has been a longstanding potato breeding objective. Genotypes with resistance to viruses (PVY, PLRV, PVS, PVX), nematodes (mainly *Globodera* and *Meloidogyne* species), bacterial wilt, and a broader spectrum of varieties tolerant to abiotic stresses like heat, drought, and saline conditions, and focus on beneficial root traits, can increase productivity and expand potato production to new areas. On the one hand, restriction on the use of pesticides and the societal push towards agricultural production with less agrochemical inputs also requires selection of pest resistant varieties. On the other hand, the increasing occurrence of periods of drought and heat that hamper production and tuber quality makes biotic and abiotic stress factors highly relevant, also, for regions like North-Western Europe. Disease resistance has been important in all breeding programs since about the 1920s. This resistance became key in breeding programs that have introduced genes from native potatoes (landraces) into commercial varieties, for instance, by CIP since the 1970s and by the DuRPh project on Late Blight control in The Netherlands from 2006 to 2015, in this case using the cis-genesis breeding approach (Haverkort et al. 2016). Such approaches should be developed further at international scale, aiming to develop collaboration and stimulate interaction and stronger knowledge exchange between researchers from potato-producing regions worldwide to make genetic advances more readily available across regions.

**Fig. 10:**
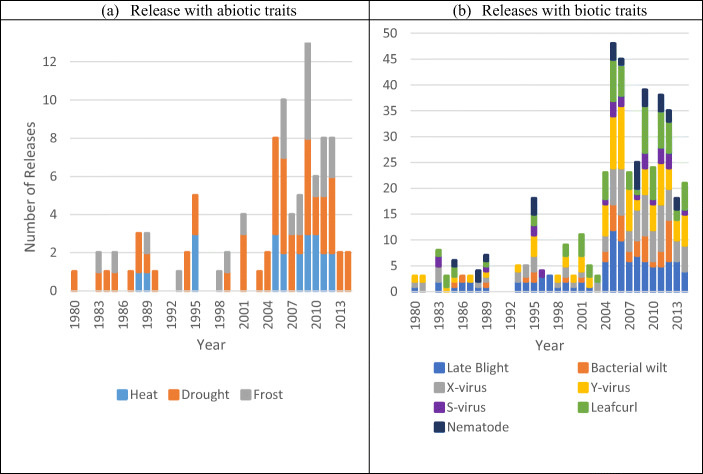
Total potato releases with abiotic and biotic tolerance/resistant traits in Asia between 1980 and 2014. Notes: Only *high* tolerance/resistant category is shown. *Medium* and *low* tolerance/resistant and *susceptible* categories are not shown. Source: Gatto et al. (2018). **a** Release with abiotic traits. **b** Releases with biotic traits

The importance and priority to develop and release varieties with high tolerance to abiotic and biotic stresses is increasing. Data from Asia serves to illustrate how a region with an exponential growth and evolution in potato-based agri-food systems has prioritized abiotic and biotic tolerance/resistance traits (Fig. 10). Starting in the early 2000s, major biotic and abiotic traits have been included into released varieties following adjustments to breeding objectives aiming to mitigate the adverse effects associated with climate change and variability, as well as production expansion.

Genetic biofortification through conventional technologies and NBTs can help to overcome micronutrient malnutrition in human populations and support the consumption of more nutritious tubers. This crop improvement approach aims to positively influence human health, as a complement to diet supplementation and food fortification. In recent years, CIP has initiated the development of Fe and Zn biofortified potatoes, under the umbrella of the HarvestPlus Program (http://www.harvestplus.org/), a global interdisciplinary alliance for developing biofortified varieties of staple crops (Amoros et al. 2020). Food security programs working to deploy biofortified crops will strongly benefit from nutritional education efforts and awareness programs considering gender roles in the beneficiary communities.

The new potato breeding strategy of using true hybrid varieties from diploid inbred parental lines is an exciting new avenue that can provide fast genetic advancement through better application of molecular approaches, which has been slow with conventional tetraploid potato breeding (Lindhout et al. 2011; Jansky et al. 2016; Ye et al. 2018). Genetically more resilient potato breeding populations can be made available to a variety of potato agri-food systems with true hybrid potato as it facilitates the incorporation of important parental genes and by exploiting heterosis by hybridization. But the future of hybrid potato breeding is both open and uncertain. This future will not only depend on the ultimate agronomic performance of hybrid potatoes but also on a range of societal actors, conditions, and developments that may steer breeding in various directions, for example, for reducing potato environmental footprint. Hybrid varieties will become available as true potato seeds and will foster new seed and cultivation system models that may vary from direct sowing by farmers to using plantlets or (mini)tubers produced in specialized nurseries. Questions remain about the implications of these various system choices for different stakeholders in the potato sector in both rural and industrial agri-food systems.

Deployment of transgenic or gene-edited potatoes with stacked R genes is another avenue with enormous potential for achieving genetic gains from the potato of the future, which is being promoted by the biotech science community, but has unfortunately been constrained by unending issues related to public acceptance of transgenic crops and issues related to the absence of legislation for commercialization of transgenic crops (Ghislain et al. 2018).

##### Seed Quality and Availability, Key to a Successful Harvest

As sustainable potato production depends on a constantly renewed supply of pest-free planting material, improving quality seed production and seed distribution is another strong avenue of research opportunity related to crop management (M). The concept of seed security contemplates different types of seed insecurity: poor seed quality, lack of availability, limited access to high-quality seed, lack of access to preferred and adapted varieties, and inefficient seed systems (FAO 2016a Bentley et al. 2018). More than 90% of seed potatoes in developing economies is produced in the farmer-based category and is considered to be of poor quality (Thomas-Sharma et al. 2015). Potato seed production systems should support the access to high-quality seed potato tubers of improved varieties by combining rapid multiplication technologies (e.g. aeroponics, sand hydroponics, or apical cuttings) with decentralized seed multiplication (e.g. promotion of quality declared seed systems (FAO 2006b; Fajardo et al. 2010). Complementing with on-farm seed maintenance (e.g. positive selection, small seed-plot technique, and improved storage) in an integrated approach will strengthen sustainability (Gildemacher et al. 2011; Schulte-Geldermann et al. 2012; Thomas-Sharma et al. 2015; Obura et al. 2016; Priegnitz et al. 2019; Priegnitz et al. 2020). Andrade-Piedra et al. (2020) emphasize the need to improve the capacity to diagnose bottlenecks on seed systems and design interventions to address them using methodologies that combine socio-economic and biophysical perspectives. In this context, improving technologies for farmer-based seed production and distribution of high-quality planting material of existing and new varieties have also the potential to reach high numbers of beneficiaries with strong impacts on poverty reduction and food availability, but this will require systematic changes to seed production regulations and their enforcement.

##### Threats to Control Emerging Pests

Emerging pests, and especially pathogens defined as causal agents of infectious diseases whose incidence increases in new or existing host populations as result of lasting changes in their epidemiology (Woolhouse et al. 2005), are serious threats to rural- and industrial-based agri-food systems. Although the type of damage caused by emerging pests is similar across agri-food systems, the societal effects are usually larger in rural systems where provision of healthy planting material and other management measures are ineffective and plant protection organizations have limited outreach. For example, ‘*Candidatus* Liberibacter solanacearum’(Lso), the causal agent of zebra chip, and its vector, the potato psyllid *Bactericera cockerelli*, are together one of the main causes of heavy yield losses in Central America (Wang et al. 2020), where rural agri-food systems are predominant. In industrial agri-food systems, zebra chip is controlled by early detection, control of weed hosts and volunteer plants, and chemical control of the vector (Munyaneza 2012), but these measures are difficult to apply in countries where rural agri-food systems are predominant and farmers rely mostly on insecticides. In pathogens such as *Phytophthora infestans* (which has been referred to as a “reemerging” pathogen, Fry et al. 2015), the causal agent of late blight, the main control measure in industrial agri-food systems is the use of fungicides and warning systems (Haverkort et al. 2009), but in rural agri-food systems, intensive use of fungicides has caused serious negative effects to farmers and their families because of the lack of protective equipment (Cole et al. 2008). New resistant varieties coupled with decision support systems and farmers’ capacity strengthening (e.g. Perez et al. 2020) are readily available options to improve late blight management in rural agri-food systems, while other technologies, such as the introgression of resistant genes from wild potato relatives into existing varieties (Ghislain et al. 2018), are expected to be available in the next years. In the case of insects, the Guatemalan potato tuber moth (GPTM), *Tecia solanivora*, illustrates how a pest can be disseminated widely. GPTM probably originated from Guatemala and is endemic throughout Central America. In 1983, the pest was unintentionally introduced into Venezuela and then invaded Colombia and Ecuador. In 2000, *T. solanivora* was introduced in the Canary Islands (Tenerife). Since then the pest has been considered as a major threat to potato throughout Southern Europe and was listed as a quarantine pest by the European and Mediterranean Plant Protection Organization (EPPO 2005). In 2014, it was finally recorded in Mainland Spain where efforts are going on to eradicate the pest (Jeger et al. 2018). At global level, learning from known cases where emerging pests caused heavy damage [e.g. potato purple top in Ecuador (Navarrete et al. 2020) or potato cyst nematodes in East Africa (Mburu et al. 2020)] as well as novel strategies such as the Global Surveillance System for plant disease (Carvajal-Yepes et al. 2019) and cropland connectivity analyses (Xing et al. 2020) will be key to ensure that the potato crop is able to adapt to emerging pests, especially in rural agri-food systems.

##### Potato Crop Management Technologies and Farming Practices to Increase Productivity and Sustainability

The highly adaptable potato can fit to many types of environments (*E*) from sea level to high mountain conditions (Haverkort et al. 2013). The adaptation of the crop depends on the genotype (*G*) but also on the crop management practices (*M*) that need to evolve according to the specific agro-ecological conditions, the socio-economic context, and the local agri-food systems. Smart agriculture is a novel avenue for resource use optimization based on new monitoring and decision support tools. Remote sensing and geographical information system (GIS) tools coupled with decision support systems (DSS) and precision agriculture (PA) technologies may contribute to increased productivity, while interaction among biophysical and social disciplines for sustainable food production intensification can strengthen resource use optimization. DSS for the application of fertilizer (N, P, K) is an example of the use of field-scale models as well as tractor, drone, or satellite embedded spectral sensors to monitor crop nutrient status to supplement plant nutrients according to inter- and within-field variability (Goffart et al. 2008, 2017). Another example used in industrial-based farming systems concerns haulm (vine) killing to induce tuber maturation (Kempenaar and Struik 2007). Classical methods for vine killing are based on mechanical/physical or chemical approaches or combinations. Recent developments in the use of embedded optical sensors on tractors or sprayers and Global Positioning Systems (GPS) enable the supply of site-specific amounts of chemicals based on quantity of biomass measured in the field (van Evert et al. 2012; Kempenaar et al. 2017). Precision haulm killing has reduced the amount of chemicals for haulm destruction up to 50% and is useful in the frame of the current and future bans of certain vine desiccants (e.g. Diquat in Europe).

Precision-farming technologies are still mainly used in industrial agri-food systems in high-income countries, but massive and varied data management could foster new models to be developed and contribute to the development of decision support tools in countries with mainly rural-based agri-food systems, using adapted hand-held or tractor-based optical sensors. For instance, CIP is adapting a DSS to manage late blight that combines host resistance and a set of decision support rules to optimize knap-sack sprayer applied fungicides for late blight control in the Andes (Perez et al. 2020).

Another example of a technology to increase productivity and sustainability is the “attract-and-kill” approach. The potato tuber moth (PTM), *Phthorimaea operculella*, originated in the tropical mountainous regions of South America. Today it has a worldwide distribution and is considered the most damaging potato insect pest in the developing world. The attract-and-kill approach has been developed to control *P. operculella* and *Symmetrischema tangolias*, the Andean potato tuber moth (APTM), under field and storage conditions. It consists of a co-formulation of the insect pest-specific sexual pheromone, which “attracts” males, and a contact insecticide at very low concentration which “kills” males getting in contact with the product. The oil formulation is applied at a droplet size of 100 μL using a special handheld applicator; it is applied at 2500 droplets/ha. It effectively reduces the male population and the number of offspring, hence controlling larvae damage in the crop. It provides pest-specific control and is harmless to natural enemies, humans, and the environment (Kroschel and Zegarra 2010; Kroschel and Zegarra 2013). In Peru, two products (AdiosMacho-Po® and AdiosMacho-St®) have been registered to be commercialized in Peru and the Andean region (Kroschel et al. 2020).

To enhance ecological sustainability, a key element is to implement management practices that increase the level of provision of ecosystem services such as natural soil fertility or biological control. Natural regulation of pests appears as an important element in sustainable potato agro-ecosystems. It has been demonstrated that the development of natural antagonist associations of the Colorado beetle, such as auxiliary insects and useful pathogens, can significantly improve the control of such a pest (Crowder et al. 2010). Research to develop biocontrol and other environmentally friendly alternatives is very active and considered to grow substantially in the coming decade, but there are still few confirmed successes from the field (e.g. use of phosphonate to control late blight, Kromann et al. 2012; Sanabria et al. 2020).

Sustainable soil management is also an important avenue for the potato crop. Regular application of organic and calcic amendments is of prime importance to maintain good soil structure in fields submitted to crop rotation including potato, and the respect of at least 4–5 seasons between potato crops should be a basic rule everywhere. Potato furrows in slope fields are a serious concern because of water runoff, soil erosion, and thereby surface water contamination with pesticides and excessive plant nutrients. The use of micro-dams in potato furrows has been demonstrated to minimize these risks and to be a cheap and easy technique to implement (Olivier et al. 2014; Sittig et al. 2020).

Specific intensification practices can be developed under specific cropping systems such as in the cereal-based systems in India through “double transplanting (DT)” of rice and planting early maturing potato between the two rice crops as a valid highly productive alternative to the traditional system for small-scale producers (Arya et al. 2015).

#### Produce Just as Much but Waste Less Through Value Chain Development and Better Post-Harvest Management

##### Inclusive Potato Value Chain Development Integrating Stakeholders

Although potato remains a staple food in rural areas in developing countries, it is also increasingly becoming a cash food for farmers in Asia, Africa, and LAC (DeFauw et al. 2012). In those regions, most potato producers are smallholders who depend strongly on agriculture, including the potato crop, for income, food security, and employment. Important factors that have spurred the interest in value chain development are changes in consumer demands for added value food products, new or emerging markets with quality and food-safety standards by private firms, and the growth of niche markets. It is necessary to create incentives for smallholders to modernize their crop management and to overcome constraints to productivity and profitability. It will contribute to have them participate more efficiently in value chains for a particular product, while ensuring the management of vertical (contracts, interaction with the value chain actors) and horizontal (producer organizations) coordination. The challenge to achieve food and nutrition security as well as prosperity for these smallholders will be achieved or lost by the way agricultural value chains are developed and managed. More effective participation in value chains will be achieved through farmer associations, better infrastructure such as storage facilities, irrigation, and better links with traders and consumers. The development of value chains that contribute to linking smallholder farmers to markets offers also new opportunities in terms of labour. As food systems evolve, the emergence of small- and medium-sized enterprises in transportation, processing, and distribution—the expanding “hidden middle” of the food supply chain—can promote inclusion of the rural poor and offer opportunities in terms of labour for a growing population of young people, women, and men that need to actively engage in income generation (de Janvry and Sadoulet 2020). While the majority of young rural women and men see their future outside agriculture, many good job opportunities on and off the farm remain linked to agriculture. In recent years, research activities to improve the efficiency of the value chain and coordination among its actors have evolved to achieve more inclusiveness in the value chain development approaches (Devaux et al. 2018). To respond to these changes and the need to make agriculture more environmentally and consumer friendly responding to societal requirements (*S*), research should be characterized by an interaction between natural and social sciences and should be demand driven considering the gender differentiated needs and challenges faced by the different value chain stakeholders. It also requires a good understanding of the market forces that determine supply and demand for any particular product and hence the profit to be made by participation in the value chain. Linked to value chain efficiency, the assessment of food losses across the value chain and the quality of marketed potatoes also requires further research efforts to optimize food availability and consumer access to quality potato products.

In countries with mainly industrial-based agri-food systems, more than one-third of all potatoes grown are manufactured into frozen products. In the USA, 85% of processed potatoes are frozen French fries (USDA 2020). In Europe, in a country like Belgium, there are two main marketing channels: potatoes intended for direct sale on fresh potato markets and the ones intended for processing, which respectively represent around 20% and 80% of national production (www.belgapom.be; www.fiwap.be). Producers are often specialized in one of these two markets, responding to the consumer requirements (*S*), because the varieties, production methods, and commercial relationships are different. The processing market has been stimulated by decades of explosive growth within the quick service restaurant industry. Processed potato products, which include frozen, chipped, and dehydrated potato, became the major drivers in the potato market, led by frozen French fries. Improvement in technical and commercial relationships and cooperation among stakeholders of the different potato value chains in Europe, from producers to processors and consumers, has been and remains crucial for the integration and successful sector development which has been amplified with the huge expansion of the processing industry in North West Europe during the last decade.

The recent COVID-19 pandemic also severely hobbled the food service sector, resulting in an abrupt slowdown in frozen products demand. It has shaken up all predictions and profoundly changed the fundamentals of the 2019 campaign in Europe. On the fresh market, it has meant a revival in terms of household consumption. For the industry, on the other hand, it has caused factories to slow down due to the very sharp decline in restaurant activity all over the world. There is some uncertainty at the demand level for the future post COVID-19 crisis, which again emphasizes the need for a united potato sector following a period of crisis and strong impacts of societal factors (*S*) at national and international levels.

##### Post-Harvest Management: Reducing Food Losses and Waste

As indicated above, another way to face the food security challenge is to produce just as much but waste less through better post-harvest management. Post-harvest management in potato, including storage, processing, and value chain efficiency, is a much larger problem than in cereals and deserves special attention. Reduction of food losses appears as a key opportunity. The basics of storage management have not changed, but the implementation and application of the basics are evolving worldwide, according to diversity in location, climate, and market criteria, that will influence storage management structures and management decisions (Olsen 2014). In developing countries, recent studies have analysed food loss across the potato value chain, as, for example, in Ecuador and Peru, by collecting qualitative and quantitative data to provide a comprehensive identification and characterization of losses. The results show that the most important losses occur in the production node, ranging from 90 to 95% of the total losses in the chain, which were on average between 8 and 9% of production along the value chain in both countries. On average farmers suffer the highest loss across the value chain, ranging between 8 and 20% of their production at or before harvest, before moving on to the next node of the chain. The main causes of losses are poor crop and harvest management practices leading to pest infested tubers, high percentage of small tubers, and weather conditions such as frost and heavy rains (Delgado et al. 2017; Velasco et al. 2020).

Potato storage facilities in industrial countries serve mainly as insurance for tuber availability for the processing industry year round, and development of sustainable technology is part of the research activities of storage facility providers. Knowledge exchange to support development of small-scale potato storage facilities in rural and low-income countries should be fostered. However, the recent ban of the effective and inexpensive sprout inhibitor chlorpropham (CIPC) in Europe by October 2020, used in high-capacity potato storage facilities and export shipments, constitutes a major technical and economic challenge for the European potato sector. It is mainly due to the high cost and limited knowledge of alternative solutions. CIPC has been used since 1959 and has enabled year-round market supply (fresh and industry) as well as exports to far-away destinations, while controlling the quality of tubers. Alternative products exist, such as mint oil, ethylene, carvone oil, and di-methyl-naphthalene, approved in Europe or 3-Decen-2-one approved only in North America. However, all of which are applied exclusively by nebulization in gaseous form, for which lots of current European storage facilities are not fully prepared. In the current scenario, the additional costs need to be borne by the producers, as the issue of competitiveness of European finished products on world markets (mainly French fries) prevents a full pass-on of the additional costs to the end consumer.

Such a story and situation illustrate the weaknesses and the limited sustainability of global and local systems based on the use of synthetically produced substances. Sustainability related to societal requirements (*S*) needs to be considered for future potato sector development, for example, in the frame of the increased banning of pesticide use in agriculture in Europe and elsewhere.

## Towards Future Potato Research for Global Food and Nutrition Security

In both rural- and industrial-based systems, innovations resulting from potato research should be incremental through a step-by-step improvement of an existing structure promoting technologies adapted to different agri-food systems. This is particularly true for smallholder family agriculture in developing countries where there is a great need to increase potato production in a sustainable way for both men and women who may face different productive challenges and opportunities. While this approach has the advantage of not destabilizing an existing system, it may also suffer from a systemic lock-in or a lack of enabling environment that keeps agriculture and agri-food systems on less efficient pathways as developed by Baret (2017). An example of lock-in is the use of pesticides and their promotion by agro-chemical companies and technical support services that influence farmers’ decision-making, restraining the use of more environmentally friendly options such as decision support tools for efficient pest control with a more rational, or even no, chemical pesticide use. The valuable use of varieties tolerant or resistant to pests can also be limited by processing companies that promote varieties for their processing characteristics giving less emphasis to their environmental footprint. In developing countries, low quality of infrastructure, weak institutions, and policies also create huge limitations to the adoption of new and more sustainable technologies.

To reach the strongest impact through potato-based agri-food systems, potato research and development efforts need to move towards cooperation between disciplines (and filling up the gaps that exist between them) to allow integration of knowledge rather than focus explicitly on single technology/solution development. In this paper, we have analysed the different components that contribute to the performance of the potato crop and sector using the performance Eq. (1) (*P* = *G* × *E* × *M* × *S*), enabling a list of key research and technology options to guide agricultural research and technology development towards sustainable intensification approaches responding to farmers’ needs both for food security and better income generation. The argument is that agricultural programs need to better integrate sustainable intensification and food security indicators considering also other dimensions such as quality, diversity of products, health impacts, and climate change effects. Multidisciplinary approaches are required to recognize and solve practical problems at the levels of the cropping system as well as the whole potato value chain to enhance potato’s contribution to sustainable agri-food systems with a better understanding of the evolving food systems that are changing rapidly with changing consumer needs and value chain requirements.

Local calibration/validation and demonstration are two essential phases towards local end-user uptake, either involving producers or extension service representatives. A key to successful adoption of innovations is the accurate definition of the needs of farmers and other stakeholders in the potato value chain considering socio-economic and stakeholder diversity. Policies and public and social investments are also required to support such actions that will enable producers to have access to innovations and to be trained in their use. In general, results show that information remains a serious constraint to farmers’ modernization. The upcoming of new communication technologies such as smartphones, expansion of mobile broadband, and access to local online platforms integrating large amounts of local data and links to digital tools offer interesting new options, but there is a need for experimentation to fully exploit the potential of information technologies particularly with young people, without exacerbating inequality gap especially in developing countries.

There is a dichotomy between potato-based rural- and industrial-based agri-food systems as illustrated in Fig. 1. Currently, “pure” rural agri-food systems may use landraces, farm-saved seed, low-cost machinery, and low amounts of agrochemicals, with potatoes being produced as staple for fresh consumption, while generating low income and low environmental footprint. In the other extreme, “pure” industrial agri-food systems may use improved varieties, certified seed, high-cost machinery, and high amounts of agrochemicals, with potatoes being produced as industrial product for processing markets, generating high income and higher environmental footprint potentially. It is also common to find combinations of both systems even at farm level, as, for example, in the Andes, with specific varieties produced for own consumption with less intensive management practices and commercial varieties produced for urban markets with different management practices. There is also a dichotomy between research activities in developed versus developing countries that highlights the need for more exchange, knowledge sharing, and collaboration. The key research and technology options proposed and analysed in this paper will require policy support and financial and non-financial services to have a chance to be promoted and used by producers of rural- and industrial-based systems. Better access to discovery research and technology needs to be provided by the potato research community through improved services at national and international levels.

As a summary of explicit while not exhaustive innovation options that could meet requirements of both rural and industrial potato-based agri-food systems worldwide, we can mention:

- Urgent supply of locally adapted varieties, tolerant/resistant to pests, drought, and heat for limited input production systems and with integrated nutritional characteristics. As a corollary, acceptation and development of recognized new breeding technologies (NBTs) to overcome public non-acceptance of transgenic crops and need to foster supporting legislation for commercializing varieties derived from NBTs.
- Enhancement of local and decentralized high-quality seed production, multiplication, and distribution systems reducing the dependency on international transports, thereby lowering long-distance transportation costs while reducing the risks associated with the spread of pests.
- Support to the selection and promotion of locally adapted, demand-led potato varieties, combined with rapid seed multiplication techniques.
- Development of pest management options for a more rational use of pesticides and alternative practices such as biological control and decision support tools, combined with integrated cropping systems for sustainable production practices including water and soil fertility management.
- Development of new storage technology and equipment for industrial-based agri-food-systems [i.e. to overcome the ban of sprout inhibitor chlorpropham (CIPC) in Europe] accompanied with knowledge exchange to enhance potato storage capacities in rural-based agri-food systems.
- Research to improve the efficiency of potato value chains and foster strengthened coordination among its actors is still required to reduce food losses, to enhance the profit to be made by the different stakeholders, and to generate attractive opportunities for the young population, without exacerbating social inequalities. This being true for both rural and industrial potato-based agri-food systems.

## Abbreviations

AFR: Africa
APTM: Andean potato tuber moth
ASA: Asia
CA: Central Asia
CIP: International Potato Center
CIPC: Sprout inhibitor and herbicide chlorpropham
CVD: cardiovascular disease
DSS: decision support systems
DT: double transplanting
EAPR: European Association for Potato Research
EPPO: European and Mediterranean Plant Protection Organization
EU: European Union
EUR: Europe
FAO: Food and Agriculture Organization of the United Nations
GDP: gross domestic product
GFI: Global Food Security Index
GHI: Global Hunger Index
GIS: global information system
GPS: Global Positioning Systems
GPTM: Guatemalan potato tuber moth
IFPRI: International Food Policy Research Institute
IPCC: Intergovernmental Panel on Climate Change
IYFF: International Year of Family Farming
LAC: Latin America and the Caribbean
NAM: North America
NARS: National Agricultural Research System
NBTs: new breeding technologies
NENA: Near East and North Africa
N, P, K: nitrogen, phosphorus, potassium
PA: precision agriculture
PGSC: Potato Genome Sequencing Consortium
PTM: potato tuber moth
QDS: quality declared seed
RDI: recommended dietary intake
SA: South Asia
SI: sustainable intensification
SSA: sub-Saharan Africa
T2D: type II diabetes
UN: United Nations
USSR: Union of Soviet Socialist Republics

## Footnotes

1 Wu et al. (2018, pg 2) define cropping intensity gap as “the amount of incremental cropping intensity that is possibly available if all croplands in a given region are fully intensively used”.
